# Changes in vitreal protein profile and retina mRNAs in *Reeler* mice: NGF, IL33 and Müller cell activation

**DOI:** 10.1371/journal.pone.0212732

**Published:** 2019-02-27

**Authors:** Bijorn Omar Balzamino, Graziana Esposito, Ramona Marino, Flavio Keller, Alessandra Micera

**Affiliations:** 1 Research Laboratories in Ophthalmology, IRCCS–Fondazione Bietti, Rome, Italy; 2 Laboratory of Developmental Neuroscience and Neural Plasticity, University Campus Bio-Medico, Rome, Italy; Peking University, Institute of Mental Health, CHINA

## Abstract

A possible link between Nerve Growth Factor (NGF) and Reelin might take place during impaired retinal development occurring in the Reelin deficient mouse model (*Reeler*). To better characterize NGF and retina impairments at the *Reeler* retina, vitreous and retina were investigated by means of protein expression and glial cell activation. *Reeler* (n = 9; RELN^-/-^) and WT (n = 9; wild-type RELN^+/+^, B6C3Fe) mice were analyzed at 14, 21 and 28 postnatal days (p). Retinas and vitreous were subjected to confocal analysis and protein array, followed by conventional analysis. A significant increase of NGF, IL33 and TIMP1, a trend to a decrease of IL12 and IL6, as well as a significant decrease of NT3 were detected in *Reeler* vitreous, particularly at p28 (p<0.05). MIP3β mRNA was decreased while IL33mRNA was significantly upregulated in *Reeler* retina. Increased number of GFAP^+^ and Nestin^+^ cells as well as upregulation of Glutamine Synthetase and Nestin mRNAs were observed in *Reeler* retinas (p<0.05). These findings extend our previous studies on *Reeler* retina showing a selective Müller cell activation. NGF and IL33 release into vitreous would suggest a local activation of Müller cells, in addition to retinal ganglion and accessory cells. Overall, the data from this experimental study would strength the potential neuroprotective role played by activated Muller cells through NGF release.

## Introduction

The absence of Reelin–a glycoprotein crucial for physiological retinogenesis–has been recently associated with changes in both Nerve Growth Factor (NGF) protein and mRNA in the retina [1–3]. NGF and Reelin have been reported to take actively part in neurogenesis and retinogenesis [4–6]. NGF has been hypothesized to interact with Reelin by modulating neuronal migration, neurodevelopment and other physiological processes in the central nervous system and retina [1,2,7]. NGF activities encompass cell proliferation, cytoskeletal reorganization, migration, differentiation, survival and/or apoptosis [4,8]. In the retina, NGF modulates retinal cell development, differentiation and functional activity, and promotes survival/recovery of Retinal Ganglion Cells (RGCs), photoreceptors and optic axons after experimental injuries as well as normal functional and anatomical development of visual acuity and binocularity [4,8–10].

The neurodegenerative process occurring in *Reeler* retina evolves through a series of changes at different cell types (neural, vascular and glial cells) and comprises several overlapping/interrelated molecular pathways [5]. Glial cell activation (astrocytes, Müller cells and resident microglia) represents a crucial step for protecting neurones from degeneration [7]. Müller cells work in concert with other glial cells and neurones to guarantee optimal development of retinal structure [9–12]. An open question regards the glia cell activation upon Reelin deficiency and the possibility for NGF to maintain retinal homeostasis via glial cell activation, as observed in other neuronal degenerating tissues [13,14].

Therefore, the aim of this study was to look for some proinflammatory/profibrogenic mediator changes in the vitreous and retina as well as glial cell activation in the retina of *Reeler* mice.

## Materials and methods

### Animals and ethical approval

Eighteen (18) animals were used for the study, including 9 *Reeler* (RELN^-/-^; 9–11 gr body-weight) and 9 WT (RELN^+/+^; B6C3Fe; 12–14 gr body-weight) mice (Charles River, Calco, Como). Experimental procedures were approved by the Ethical Committee of Tor Vergata University (Rome, Italy), according with ethical standards stated in the Declaration of Helsinki and the ARVO Statement for the Use of Animals in Ophthalmic and Vision Research. All the steps in the procedure were in compliance with the directive 2010/63/EU guidelines, under the authorization provided by the Italian Ministry of Health. All efforts were made to minimize suffering.

All analytic and molecular grade reagents were purchased from Carlo Erba (Milan, Italy), Euroclone (Milan, Italy) and Sigma (Milan, Italy), otherwise specified in the text. Daily produced MilliQ RNAse-free water was provided for biochemical and molecular purposes (Direct Q5; Millipore Corp., Billerica, MA).

### Experimental procedure: Vitreous and retina

At postnatal day (p) p14, p21 and p28, mice were anaesthetized by intraperitoneal injection of 2 mg/mL ketamine (0.2 mL/10 gr body-weight; Ketavet, Gellini Pharmaceutics, Italy) and 0.23 mg/mL medetomidine (0.24 mL/10 gr body-weight; Domitor, Orion Corp., Espoo, Finland) mixture. Sampling was carried out under a dissecting microscope (SMZ645; Nikon, Tokyo, Japan) equipped with cold-light optic fibers (PL2000 photonic; Axon, Vienna, Austria), as previously reported with slight modifications [2]. A corneal incision was produced and lens, retina and vitreous were collected in a microvial with separating membrane. Centrifugation (13.000rpm/15min) was performed to detach vitreous from retina and lens. Vitreous (left/right eyes) and retina (right eye) were appropriately stored for biochemical and molecular studies. Other retinas (left eye) were used for immunofluorescent analysis.

Vitreous and retina (n = 3/time-point; *Reeler* and WT mice) were diluted / extracted in modified RIPA Buffer (50mM Tris-HCl, 150mM NaCl, 1% Triton-X100, 5mM EDTA, 100mM NaF and 1mM PMSF; pH 7.5) and finally sonicated (VibraCell Sonics, Inc., Newtown, USA), according to a standard procedure [2]. Total proteins were quantified with Nanodrop Spectrophotometer (A1000, Celbio, Milan, Italy).

### Protein array

A customised chip-based array was used to quantify inflammatory/profibrogenic factors in vitreous and retinal lysates, between a list of potential candidates (G-series arrays; Ray Biotech, Norcross, CA). Each glass-slide comprised 14 identical sub-arrays containing 50 factors (antibody spots in duplicate) retrospectively selected by literature search [15–18]. *Reeler* and WT samples were processed simultaneously. Briefly, normalized protein extracts (50μg total protein; 70μL per well) were diluted in appropriate buffer and hybridized in sub-arrays. Washing, detection and labelling steps were performed according to the manufacturer’s recommendation. Spin-dried slides were scanned in a GenePix 4100A Microarray platform (Molecular Devices LLC, Sunnyvale, Silicon-Valley, CA). Capturing conditions and image digital acquisitions were done as previously reported [19]. Images were uniformly adjusted for size, brightness, contrast and chip-to-chip comparisons by the software and provided as 8-bit Tiff format (Axon GenePix Pro 6.0.1.25 software; Molecular Devices). Inter-assay normalization was guaranteed by the presence of multiple internal controls for each sub-array. The sensitivity range was 3.8–56 pg/mL, as provided by the manufacturer. Microarray data are available in the ArrayExpress database (http://www.ebi.ac.uk/arrayexpress) under accession number E-MTAB-7622.

### NGF ELISA

Samples were further diluted 1:2 in assay diluent (10mM PB, 150mM NaCl, 0.5% BSA, 0.1% Triton X100 and 1x protease inhibitor cocktail (Pierce—Thermo Fisher Scientific Inc.; Waltham, MA USA); Ph 7.5). Briefly, 96-well Maxisorp enzyme-linked immunoassay plates (Nunc, Roskilde, Denmark) were precoated with monoclonal anti-βNGF antibodies (0.4 μg/mL; MAB256; R&D Systems Inc, Minneapolis, Minnesota, USA). Standards (0.15 pg/mL to 1 ng/mL βNGF; Alomone Labs, Jerusalem, Israel) and samples were incubated at 4°C for 18 hours. ELISA was developed by using polyclonal biotinylated anti-NGF antibodies (0.15 μg/mL, BAF256; R&D), horseradish peroxidase streptavidin (1:300; DY998, R&D) and the ready-to-use TMB substrate (eBioscience, San Diego, CA, USA). Under these conditions, no cross reactivity with Brain Derived Neutrophic Factor (BDNF) or Neutrophins 3/4/5 was observed. The colorimetric signals (Optic Density, OD; λ450-570nm) were quantified using the Sunrise plate reader (Tecan Group Ltd., Männedorf, Switzerland) and the related mean values (pg/mL) were produced according to 3^rd^ grade polynomial standard curve and normalized to total protein content (A280; Nanodrop).

### Total RNA extraction, cDNA synthesis and real-time PCR analysis

Total RNA was extracted from retinas according to the TRIfast procedure (EuroClone) and rehydrated in 10μL fresh RNAse free MilliQ water, before treating with RNase-Free DNaseI (2U/μL; AM-1907; Turbo DNA free kit; Ambion Ltd., Huntingdon, Cambridgeshire, UK). Quantity and purity (>1.8; A280 program, Nanodrop) as well as sign of RNA degradation (1% agarose gel analysis) were checked. cDNAs were generated from normalised templates (1μg RNA) (ImProm-II Reverse Transcription System; Promega Corp., Madison, USA) in a One Cycler programmable thermocycler (PeqLab Biotech, Erlangen, Germany) and amplified using the SYBR Green PCR core reagent kit (Applied Biosystems, Foster City, CA) in Eco Real Time PCR thermocycler equipped for 48-well plate (Illumina, MA, USA). Specific primers and amplification procedure are summarised in **Table 1**. Samples were amplified in duplicate and in parallel with negative controls (either without template or with mRNA as template). Real Cycle numbers (Cn) were recorded and normalized for referring genes run in parallel (nCq = Cq_target_—Cq_referring_). The Cq averages were calculated from these replicates and showed as expression ratios (log2-scale) of a normalized target gene REST analysis [20].

**Table 1:** Primers for real time PCR.

TARGET	Sequence^#^	Amplicon Length
**IL33**	F: 5’-TGAGTCTCAACACCCCTCAA-3’	169
**MIP3α**	F: 5'-CTCCTGGCTGCTTTGATGTC-3'	151
**MIP3β**	F: 5’-GTGCCTGCTGTAGTGTTCACC-3’	133
**GS**	F: 5’-GCCCCCTATCAAGGAACTT-3’	173
**Nestin**	F: 5’-GGCCATGACTCTGACCTCTC-3’	190
**GAPDH**	F: 5’-GTGGACCTCATGGCCTACAT-3’	117

Amplification procedure: initial hot start activation (95°C/15min) followed by 39 cycles of Denaturation (94°C/10s) / Annealing (58°C/15s)/Extension (75°C/10s) and melting curve generation (55°C—95°C with one fluorescence reading every 0.5°C).

^#^Forward sequences are reported.

### Epifluorescent analysis and integrated optical densitometry

Post-fixed and cryoprotected eyes were quickly frozen in dry-ice, embedded in OCT medium (TissueTek; Leica, Heidelberg, Germany) and sectioned (CM3050 cryostat; Leica Microsystems, Rijswijk, Netherland). Serial sections (10μm) were placed on BDH slides (Milan, Italy) and stored at -20°C. Antigen retrieval (0.05% trypsin-EDTA solution, 2min) and blocking/permeabilizing (1% BSA and 0.5% Triton X100 in PBS, 15min) steps were performed. The specific antibodies were anti-GFAP (1:100; G3893; Sigma-Aldrich, St. Louis, MO, USA; 1:50; #AB5804; Merck-Millipore, Darmstadt, Germany), anti-Nestin (1:500; NB300-266; Novus Biologicals, Littleton, CO, USA) and anti-CD45 (1:100; sc-1178; Santa Cruz Biotech, Santa Cruz, CA, USA). The secondary antibodies were Cy2 (green) and Cy3 (red) conjugated species-specific antibodies (1:1000; donkey; Jackson ImmunoResearch, Europe Ltd, Suffolk, UK). DAPI was used for nuclear counterstaining (D9542; Sigma-Aldrich, St. Louis, MO, USA). Negative controls (isotypes) were carried out in parallel with the omission of primary antibodies and used for appropriate background subtractions. Serial images were acquired by NIS software connected to Epifluorescent direct microscope (Eclipse N*i*; Nikon, Tokyo, Japan).

### Data management and statistical analysis

Graphics were assembled by using the Prism5software (GraphPad software Inc., La Jolla, CA, USA), while statistical analysis was carried out with the StatView II software for PC (Abacus Concepts. Inc., Barkley, CA, USA). A p<0.05 value was considered statistically significant.

For chip-based array, the Fluorescent Intensity (FI) values were obtained by subtracting the background signal (GenePix Pro 6.0.1.25 software–Molecular Devices). Single FI values were entered into a Microsoft Excel database (Microsoft, Redmond, WA, USA) and duplicate spots outside the 10% coefficient of variability were refused from the statistical analysis. FDR value of 0.01 was set. FI averages (means±SD) were calculated from replicates (2 spots) of not-pooled samples. The following cut-offs were used: Fold Changes ≥ or ≤ 2 (FC; herein defining a given candidate factor with respect to control) and pValues ≤ 0.001 for multiple testing (p = 0.05/50 targets; two tails T-test followed by Bonferroni Correction).

For Integrated optical Density (IntDen), the 8-bit TIFF saved digital images (512x512 or 1024x1024 dpi; n = 5 sections/slide; x40/dry 0.75 DIC M/N2) were subjected to single analysis with the ImageJ v1.43 (NIH-http://rsb.info.nih.gov/ij/). IntDen data (mean±SD per retina field) were calculated, grouped and subjected to statistical analysis.

## Results

### A specific protein profile characterizes vitreous at p14, p21 and p28

**Fig 1:**
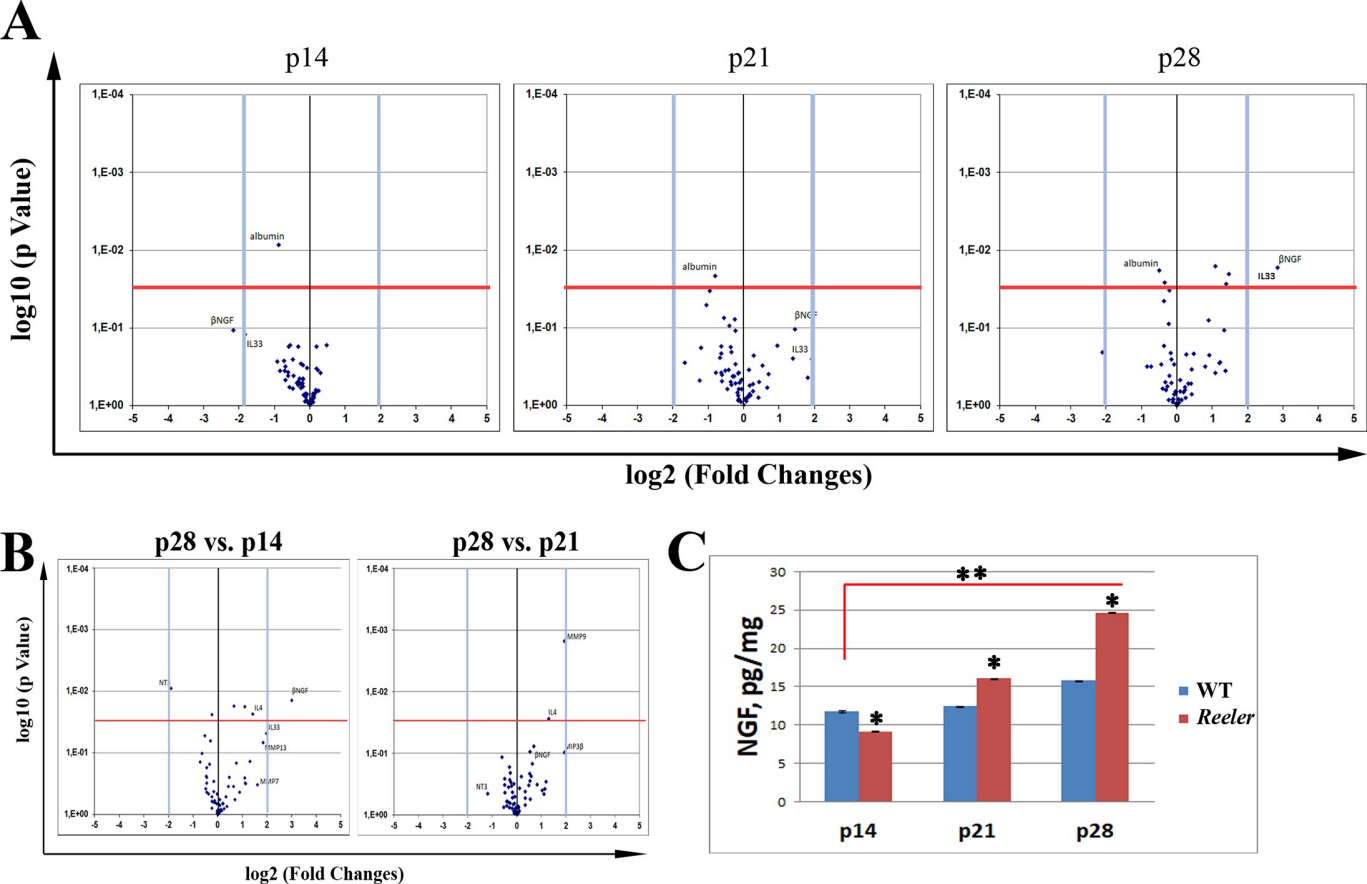
Time-dependent protein profile for *Reeler* vitreous (p14—p21—p28). Proteins were extracted from not-pooled vitreous samples and subjected to protein array analysis, as described in MM section. (**A**) Volcano Plots representative for p14 (left), p21 (middle) and p28 (right) *Reeler* vitreous as compared to WT. (**B**) Volcano plot representative for p28 *Reeler* vitreous with respect to p14 (left) and p21 (right) *Reeler*. Data are log2 FC (Fluorescent Intensity) values as provided at the end of Genepix analysis. Fold changes (±2 FC) and pValues (p< 0.001) were used as initial cut-offs (two-sided unpaired t-test statistical comparisons). (**C**) As corroborated by NGF ELISA, note the time-dependent changes of NGF expression in *Reeler* extracts at p14, p21 and p28. Significant differences between subgroups are shown. (**p<0.01; ANOVA, mean±SEM and pg/mg for total protein; *Reeler vs*. WT).

To characterize the pro-inflammatory/fibrogenic profile, both vitreous and retinal extracts were analyzed for protein array (see above). The Volcano plots in **Fig 1A** highlight the changes of interest as detected at all time-points investigated. At p14 (left panel), changes for NGF (-4.45 FC; p>0.05) and IL33 (-3.57 FC; p>0.05) expression were observed in *Reeler* vitreous with respect to WT. At p21 (middle panel), MIP3β (-3.15 FC; p>0.05) and MIP3α (-2.36 FC; p>0.05) were decreased while MMP7 (3.79 FC; p>0.05), MMP13 (3.53 FC; p>0.05), NGF (2.73 FC; p>0.05) and IL33 (2.60 FC; p>0.05) were increased in *Reeler* vitreous with respect to WT. At p28 (right panel), NGF (7.23 FC; p<0.001), IL33 (4.96 FC; p<0.001) and TIMP1 (4.79; p<0.001) continued to be increased in *Reeler* vitreous while NT3 (-4.32 FC; p<0.05) was significantly decreased in *Reeler* vitreous as compared to WT. A trend to an increase was observed for MMP9 (2.76 FC; p>0.05) and as well as for sTNF-RI/II (2.12 and 2.11; p>0.05). Significant results are listed in **Table 2**.

**Table 2:** Sketch of significant inflammatory/profibrogenic mediators in p28 *Reeler* vitreous.

Mediator	Function	FC (Sign)	Cell origin in the retina	[ref]
**βNGF**	Growth factor	7.23 (*)	**all cells**	[1,2,50]
**IL33**	Pro-inflammatory and pro-fibrogenic cytokine	4.96 (*)	**glial cells**	[35,40]
**TIMP1**	Metalloproteinase inhibitor	4.79 (*)	**glial cells**	[52]

Values obtained from protein chip array showing the fold changes (FC) in the expression of some inflammatory/profibrogenic mediators at p28 *Reeler* retina as compared to WT. Significant values (Sign) are labeled by asterisk (*, p<0.05) or ns (not significant).

As shown in **Fig 1B**, the comparison between p28 and p14 *Reeler* protein profiles confirmed the significant increase of NGF expression in the vitreous. Interesting, the NGF increase was time-point dependent, as validated by ELISA (**Fig 1C**; Particularly, NGF levels were significantly increased at p28 (24.663±0.045 *vs*. 15.770±0.050 pg/mL; p<0.01, *Reeler vs*. WT) with respect to p21 (16.049±0.034 *vs*. 12.456±0.016 pg/mL; p<0.05, *Reeler vs*. WT), both as compared to related WT counterparts. Only a trend to a decrease was detected for NGF at p14 (9.785±0.063 *vs*. 11.475±0.094 pg/mL; p>0.05, *Reeler vs*. WT).

### IL33, MIPs, Glutamine Synthetase (GS) and Nestin mRNAs’ expression in *Reeler* retinas

**Fig 2:**
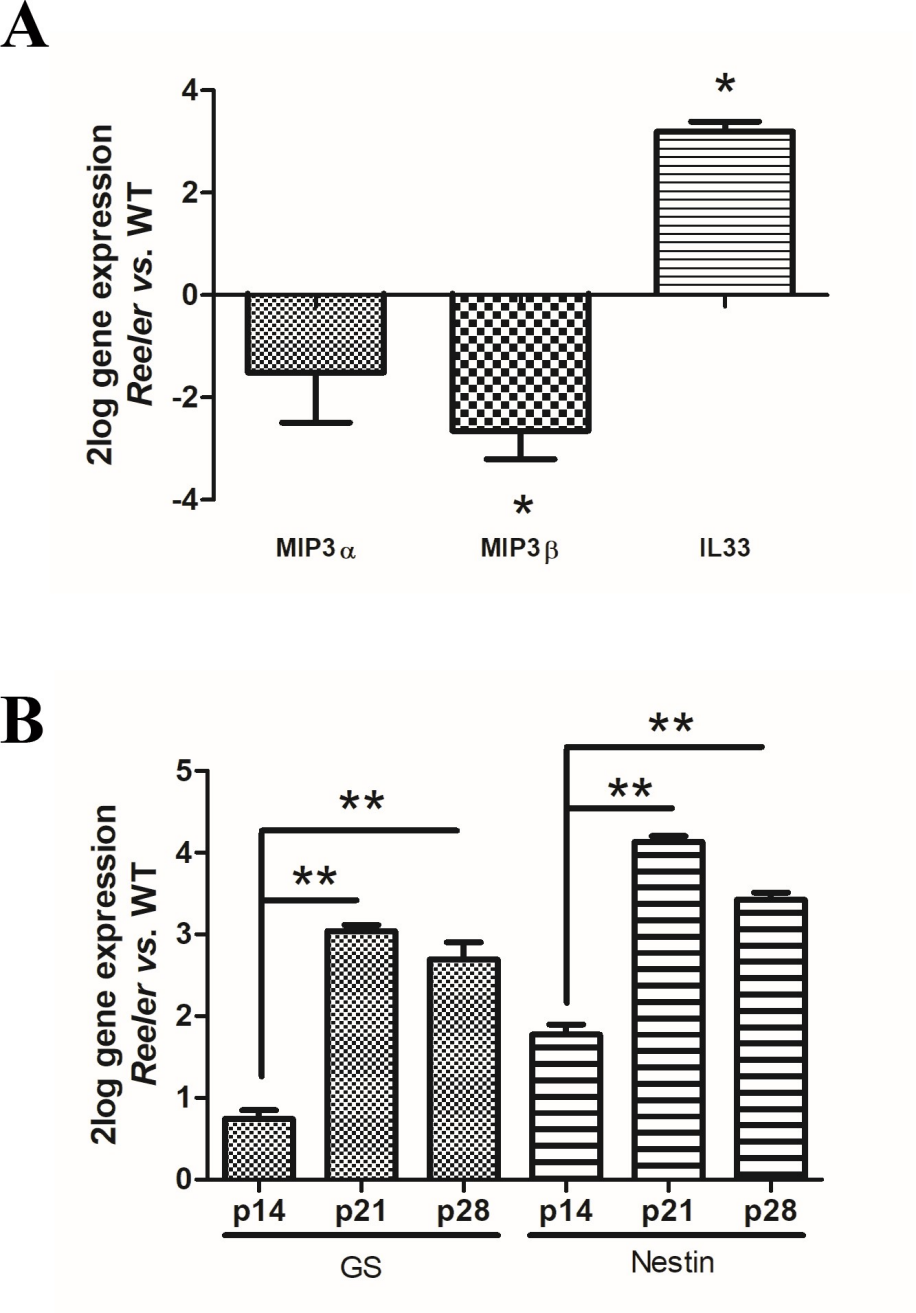
IL33, MIPs, Glutamine Synthetase (GS) and Nestin mRNA expression in *Reeler* retinas. Total RNA was extracted from not pooled retinas and used to generate cDNA for real time PCR analysis. (**A**) Histogram showing a downregulation for MIP3α and MIP3β mRNAs and an upregulation for IL-33 mRNA in *Reeler* retinas, as compared to WT ones. (**B**) Histogram showing mRNA expression changes for GS and Nestin at all time-points investigated, representative of a local Müller activation. Significant differences between subgroups are shown as *p<0.05 and **p<0.01; ANOVA-REST coupled analysis). Data are 2log gene expression (mean±SEM, *Reeler vs*. WT).

MIPs and IL33 changes were verified p28 *Reeler* and related WT retinal extracts by real time PCR. As shown in **Fig 2A**, a significant upregulation of IL33 transcript was detected in *Reeler* retinas (3.125±0.227 _2log-ratio_; p<0.01, *Reeler vs*. WT). By contrary, MIP3α (-1.512±0.691 _2log-ratio_) and MIP3β (-2.678±0.335 _2log-ratio_) transcript downregulations were observed in *Reeler* retinas (p>0.05; *Reeler vs*. WT).

Müller cell activation was investigated by means of GS and Nestin mRNA expression. A significant upregulation of mRNAs’ specific for GS (p14: 0.743 ± 0.103 _2log-ratio_; p21: 3.041 ± 0.075 _2log-ratio_ and p28: 2.694 ± 0.210 _2log-ratio_; p<0.05, *Reeler vs*. WT) and Nestin (p14: 1.774 ± 0.124 _2log-ratio_; p21: 4.130 ± 0.072 _2log-ratio_ and p28: 3.423 ± 0.085 _2log-ratio_; p<0.01, *Reeler vs*. WT) was observed as displayed in **Fig 2B**.

### Reactive müller cells populate *Reeler* retinas at p28

**Fig 3:**
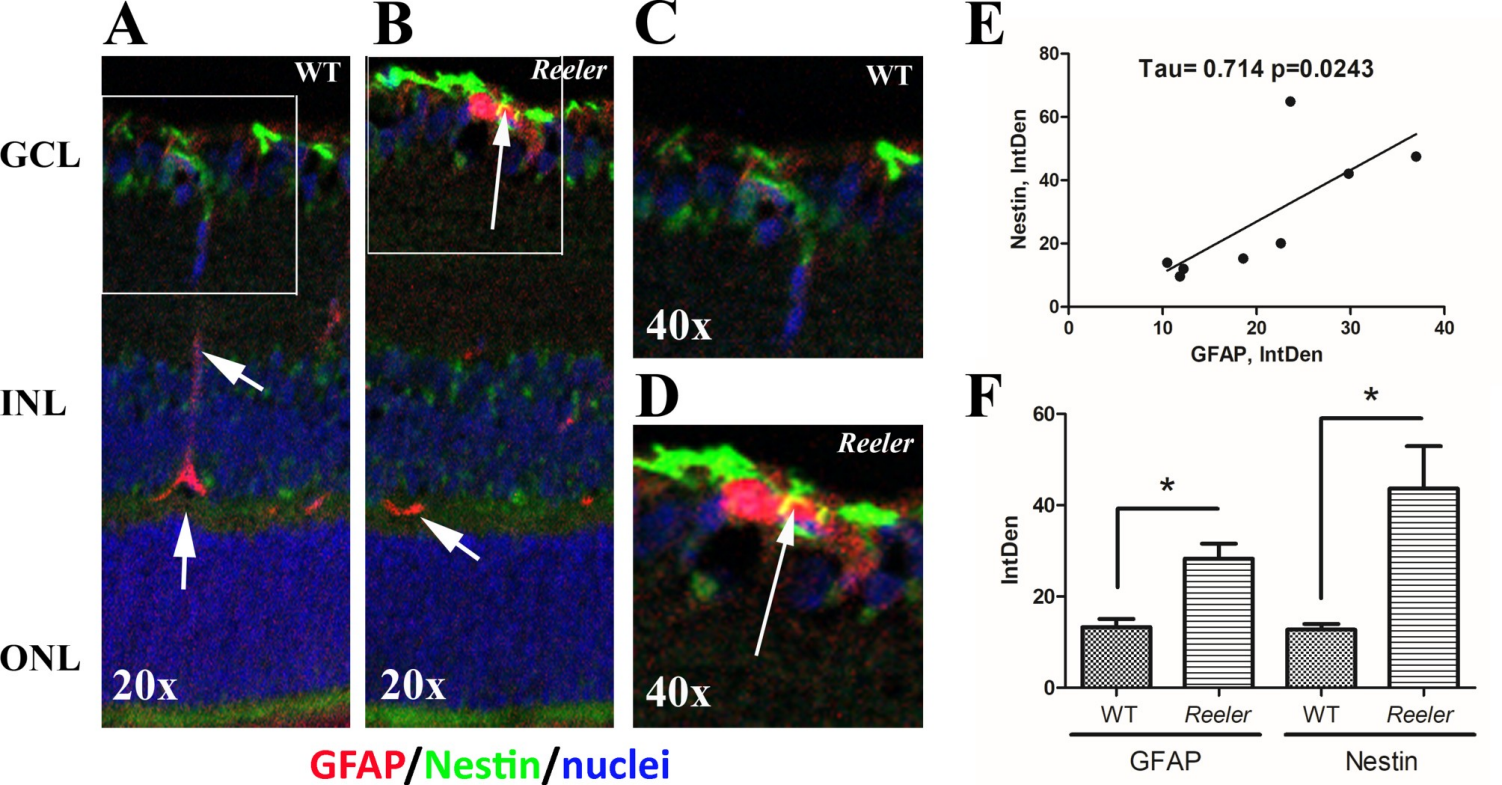
GFAP and Nestin immunoreactivity in retinal sections. Epifluorescent acquisition of p28 *Reeler* and WT retinas. (**A-D**) As compared to WT, both GFAP (red) and Nestin (green) immunoreactivities were highly visible in *Reeler* retinas (**AB**, GCL). Arrows point at yellow immunoreactivity in the white frame (**B**) indicating GFAP and Nestin co-expression in cells having long-filaments (activated Müller cells), as compared to wild type (arrowheads in **A**). Magnifications are provided in **D** and **C** respectively. Nuclei were DAPI counterstained (blue). (**E**) Scatter plot showing a positive correlation between GFAP and Nestin IntDen values. Both tau and p values obtained with Kendall analysis are reported in the panel. (**F**) Histogram representative of GFAP and Nestin IntDen analysis (mean±SEM) in both *Reeler* and WT retinas. Note the increased values for *Reeler* with respect to WT (*p<0.05, ANOVA analysis). Abbreviations: GCL, Ganglion Cellular Layer; INL, Inner Nuclear Layer; ONL, Outer Nuclear Layer; IntDen, Integrated Optical Density. Magnifications: x200 (**AB**) and x400 (**CD**).

Serial sections were immunostained for GFAP, Nestin and CD45 (leukocyte common antigen expressed by T/B lymphocytes, granulocytes, monocytes/macrophages and retinal microglia) specific antibodies [21]. Vimentin and GS were not investigated by immunofluorescence. An increased immunoreactivity for GFAP (red) was observed in *Reeler* retinas (**Fig 3B**) with respect to WT (**Fig 3A**), as merged on a blue nuclear staining. Increased GFAP immunoreactivity was mainly localized at the Ganglion Cellular Layer (GCL), although a slight immunostaining was also detected at the inner nuclear layer (INL; see arrows). A co-expression of GFAP (red) and Nestin (green) was observed in *Reeler* retinas, as pointed by arrow in the white frame (**Fig 3B**), with respect to WT retina (**A**). This GFAP-Nestin co-expression in *Reeler vs*. WT retinas is better shown in related magnifications (**Fig 3D** *vs*. **3C**). A positive correlation between GFAP and Nestin was provided by Kendall analysis (**Fig 3E**). The histogram showing the densitometric analysis for GFAP (28.25±3.32 *vs*. 13.29±1.30 IntDen; p<0.05, *Reeler vs*. WT) and Nestin (43.66±9.24 *vs*. 12.73±1.23 IntDen; p<0.05, *Reeler vs*. WT) is displayed in **Fig 3F**. Either alone or in combination with GFAP, not significant changes were observed for CD45 immunoreactivity between *Reeler* and WT retinas (18.19±3.38 vs. 17.30±1.71 IntDen; p>0.05, Reeler vs. WT).

## Discussion

Herein, we show for the first-time changes in vitreous proteins and the presence of reactive Müller cells in the retinas of *Reeler* mice. The results are below discussed.

Our previous studies highlighted the overexpression of NGF and NGF receptors (mRNA/protein) in *Reeler* retina at p14, p21 and particularly p28, postulating a potential compensatory NGF activity in Reelin deficiency [1,2]. Albeit Reelin deficient retina does not show any crucial inflammatory state, the presence of several cell-to-cell and cell-to-mediator events cannot be excluded during the neurodegenerative process that might include the expression and/or modulation of gene and related products [1,3].

Therefore, to better characterize retinal microenvironment and provide further information on NGF interplays in Reelin-impaired retinogenesis, possible mediator changes were investigated in vitreous and retina from *Reeler* and WT mice, at the same time-points (p14-p21-p28).

First, increased expression for NGF, IL33 and TIMP1 proteins as well as a trend to an increase for MMP9 and TNFR-I/II were quantified in p28 *Reeler* vitreous. Vitreous represents a precious tool to support retinal disorder management (personalized medicine), being a reservoir of active mediators and an indicator of the underneath suffering retina, especially as vitreal reflux [16,22,23]. Vitreous is a normal clear jelly-form ocular fluid composed of collagens, sulphated proteoglycans and hyaluronan [24]. Widely, pathological vitreous is filled of inflammatory/angiogenic factors (cytokines, chemokines, growth factors and enzymes of extracellular matrix), depending on the grade of retinal inflammation/degeneration conditions or even microcirculation / systemic influences [25,26]. The detection of changes in NGF, IL33 and TIMP1 as well as MMP9 and soluble TNFR-I/II proteins in *Reeler* vitreous suggest the presence of a “low” proinflammatory/profibrogenic profile, in line with previous studies [27]. This *Reeler*-associated protein profile might be consistent with the degenerative condition due to neuron-to-neuron impairments, morphological changes and reduced retinal cell migration (rod bipolar cells) [5,28]. The overexpression of NGF in *Reeler* vitreous at p21 and p28 is in line with our previous studies on *Reeler* retina and other systems [1,2,9]. With respect to MMP9 and TIMP1 changes in *Reeler* vitreous, a possible explanation might lay in a steady MMP9/TIMP1 ratio known to be crucial for the extracellular matrix stability and particularly during development and synaptic plasticity [29]. Furthermore, the increased TIMP1 levels in *Reeler* vitreous might be an attempt to counteract the lack of Reelin by inhibiting the MMPs that are involved in the cleavage of Reelin glycoprotein, as observed in other experimental models [30]. Although needing further investigations, the increased MMP7 and MMP13 levels might participate in tissue remodeling and protective effects [30]. Although not significant, the overexpression of TNF-RI/II might be consistent with the recent evidence that a soluble counterpart exists for a number of signaling receptors (for review, see [31]). Soluble receptors participate to cell apoptosis, survival, proliferation and differentiation, and particularly the “receptor-shedding” mechanism seems to modulate or inhibit ligand activity/function by preventing the interaction with proper cellular targets [32].

The consistent overexpression of vitreal IL33 at p28 appears of great interest. A possible explanation might be found in the pathogenic IL33 route during the impaired retinogenesis occurring in Reelin-deficient cells/retina. Liu and coworkers associated IL33 human expression with retinal lesions occurring in Age related Macular Disease as well as with the loss of photoreceptor and retinal ganglion cells in the experimental model [33]. In other systems, the constant “bright light exposure”—a condition that can trigger photoreceptor lost—was associated with IL33 overexpression and explained as an attempt to balance the minor cell damage and glial cell activation [34,35].

Pathological vitreous retains most of retina-derived products, representing an indirect source of retina microenvironment information in vitreoretinal practice [25]. The majority of vitreous-detectable soluble proteins originate from vitreous itself (hyalocytes) or the surrounding tissues (ciliary body and retina), while albumin might be also of plasma source [26]. Therefore, a molecular approach was performed on retinal total RNA extracts to validate some protein changes quantified in the vitreous. While the biomolecular expression of NGF in the retina was previously investigated, the herein observed IL33mRNA overexpression in retinal extracts corroborated the biochemical data on vitreous, suggesting a potential IL33 driven activity on *Reeler* retinal cells. The significant downregulation of MIP3α and MIP3β mRNAs in *Reeler* retinas can sustain the absence of inflammation and local macrophage recruitment [1,2,36]. The observation of increased IL33mRNA expression in the retina might provide explanation for increased IL33 in the vitreous and suggest *in situ* glial cell (Müller cell) activation. In rodent and human retina, IL33 (IL1 family) is locally expressed (epithelial, endothelial, glial and Müller cells), modulates immune cells (T-helper, macrophages, eosinophils and mast cells), recruits innate cells (neutrophils, macrophages, dendritic cells and eosinophils), induces hematopoietic stem / progenitor cell mobilization and even triggers the Th2-cytokine pattern [37–40]. Of interest, Müller cells (i.) are crucial in maintaining laminar structure, neuronal survival, metabolic homeostasis and retinal regeneration; (ii.) participate in reactive gliosis in response to injury and interesting (iii.) synthesize/release IL33 *in vivo* under stressing conditions [35,41].

To verify the presence of reactive Müller cells, we checked for GFAP and GS mRNAs in retinal extracts as well as GFAP and Nestin immunoreactivity in retina sections, all known as Müller cell markers in aged, damaged, injured and stressed retinas [42]. Merely, GS and Nestin represent well-known marker for Müller cell identification *in situ* while GFAP represents a marker of activated Müller cells [43]. A significant increase of GS mRNA expression and immunoreactivity (cell number and protein accumulation in Müller cells) occurred in *Reeler* retinas. Since GFAP is a marker of both astrocytes and activated Müller cell, the observation of increased GFAP-positive and GFAP/Nestin-positive cells would suggest the presence of astrocytes and activated Müller cells [44]. The absence of GFAP/CD45-immunoreactivity would exclude the microglia involvement [45]. Newly generated GFAP-positive glial cells (gliogenesis), as well as their local differentiation into astrocytes, have been previously reported in *Reeler* mice and this increase might reflect the “misorganization” of retinal layers [5,28]. According to literature, gliosis and reactive Müller cells occur in response to injury and/or upon cytokine as well as growth factor stimulation (VEGF, NGF, …) [41]. On the other hand, both astrocytes and Müller cells are source of NGF and in turn can utilize NGF by means of trkA^NGF^ expression, as reported in human retinal diseases and experimental models [43,46]. Therefore, the increased NGF expression described in *Reeler* retina and the observation of increased NGF level in *Reeler* vitreous might be view as a direct product of astrocytes/Müller cell activation, in addition to the neuronal task [1,2,47]. Corroborating data indicate that Müller cells are active players in retinal injury and chronic inflammatory/autoimmune mediated retinal disorders [48]. Long-term gliosis releases a plethora of inflammatory cytokines that lead to secondary injury, exacerbating the inflammatory reaction [47]. Local reactive gliosis might be view as an attempt to protect neuronal tissue from further damage [49].

## Conclusions

Taken together, the finding herein reported reinforce the observation that NGF might be a compensatory effector of Reelin deficiency during postnatal brain and retinal development. NGF appears to function as an intrinsic determinant of cell migration, acting as a compensative regulator of Reelin expression [50,51]. Cumulating, the NGF and IL33 overexpression in *Reeler* vitreous and the Müller cell activation in the retina would strength the presence of a protective mechanism to prevent damage and/or promote tissue repair. According to literature, the proposed (neuro)-protective attempt might be not enough due to the activation of other proinflammatory target genes (cytokines, chemokines, growth and neurotoxic factors) [2,9–10,18,25,33,41,51]. On the other hand, the overexpression of NGF, IL33, TIMP1, MMP9 and sTNFR-I/II factors to counteract/delay failures in retinogenesis and the potential NGF modulation of Müller cell activation represent interesting points that deserve further investigation for alternative strategies not restricted to reelin deficiency.
